# RC-Net: Regression Correction for End-To-End Chromosome Instance Segmentation

**DOI:** 10.3389/fgene.2022.895099

**Published:** 2022-05-18

**Authors:** Hui Liu, Guangjie Wang, Sifan Song, Daiyun Huang, Lin Zhang

**Affiliations:** ^1^ School of Information and Control Engineering, China University of Mining and Technology, Xuzhou, China; ^2^ Engineering Research Center of Intelligent Control for Underground Space, Ministry of Education, China University of Mining and Technology, Xuzhou, China; ^3^ Department of Biological Sciences, AI University Research Center, Xi’an Jiaotong-Liverpool University, Suzhou, China

**Keywords:** karyotype analysis, chromosome abnormalities, instance segmentation, end-to-end, correction

## Abstract

Precise segmentation of chromosome in the real image achieved by a microscope is significant for karyotype analysis. The segmentation of image is usually achieved by a pixel-level classification task, which considers different instances as different classes. Many instance segmentation methods predict the Intersection over Union (IoU) through the head branch to correct the classification confidence. Their effectiveness is based on the correlation between branch tasks. However, none of these methods consider the correlation between input and output in branch tasks. Herein, we propose a chromosome instance segmentation network based on regression correction. First, we adopt two head branches to predict two confidences that are more related to localization accuracy and segmentation accuracy to correct the classification confidence, which reduce the omission of predicted boxes in NMS. Furthermore, a NMS algorithm is further designed to screen the target segmentation mask with the IoU of the overlapping instance, which reduces the omission of predicted masks in NMS. Moreover, given the fact that the original IoU loss function is not sensitive to the wrong segmentation, *K*-IoU loss function is defined to strengthen the penalty of the wrong segmentation, which rationalizes the loss of mis-segmentation and effectively prevents wrong segmentation. Finally, an ablation experiment is designed to evaluate the effectiveness of the chromosome instance segmentation network based on regression correction, which shows that our proposed method can effectively enhance the performance in automatic chromosome segmentation tasks and provide a guarantee for end-to-end karyotype analysis.

## Introduction

### Motivation

Chromosomes are essential carriers for genetic information, and their abnormalities may result in congenital genetic diseases (Schrock et al., 1997). Healthy human cells contain 46 chromosomes, including 22 pairs of autosomes and 1 pair of sex chromosomes (two X sex chromosomes for women and one X and one Y chromosome for men) (Tjio, 1956; T. Arora and Dhir, 2016). Chromosome karyotype analysis, as shown in Supplementary Figure S1, can be achieved mainly by cell culture, shooting and imaging, image segmentation followed by chromosome identification (Altinordu et al., 2016). Thus, the karyotype analysis has become a common and significant method for prenatal diagnosis, genetic disease diagnosis, and screening (Garimberti and Tosi, 2010; Jahani et al., 2011; Abid and Hamami, 2018). Furthermore, the accuracy of chromosome image segmentation directly determines the accuracy of subsequent chromosome classification and abnormality identification, which makes segmentation the primary task of the karyotype analysis (Wang, et al., 2021). However, as a flexible substance (Almagro, et al., 2003), even chromosomes with the same number will show different curved shapes in different photos, and clustering will occur due to the contact and overlap of chromosomes (Somasundaram, 2019). At present, the segmentation of overlapping chromosomes is mainly done manually by cytologists, which relies heavily on the operator’s experience. Thus, it is time-consuming, labor-intensive, and error-prone. Thus, how to automatically and effectively segment a single chromosome and improve segmentation accuracy has become a critical topic in karyotype analysis (Sharma et al., 2017).

### Related Work

Traditional automatic chromosome segmentation methods are mainly based on geometric morphology (Somasundaram and Nirmala, 2010; Balaji, 2012; Sreejini et al., 2012; Balaji and Vidhya, 2015; Nair et al., 2015; Pravina, 2015; Vijayan et al., 2015; Li et al., 2016) and threshold (Ji, 1994; M.F.S. Andrade, et al., 2018; Ji, 1989). The segmentation of overlapping chromosomes is achieved by extracting features such as pits, tangent points, and refined skeletons of overlapping chromosomes. Somasundaram et al. (2014) first used the multi-object geodesic contour method to separate individual chromosome. For overlapping chromosomes, the curvature function was first used to identify the cutting points on the image. Then, the obtained cutting points were used to draw hypothetical lines on the overlapping areas. Finally, the non-overlapping chromosomes were segmented. Yilmaz et al. proposed a method of thresholding and watershed segmentation to separate chromosome clusters, calculate the tangent points of the chromosome clusters through the curvature function, and segment the overlapping chromosomes through the optimal geodesic path between the tangent points (Yang and Kruggel, 2008). Minaee et al. (2014) first extracted the outlines of overlapping chromosomes. They then applied VAMD (Variations in the Angle of Motion Direction) and SDTP (Sum of Distances among Total Points) to extract the tangent points. The segmentation effect for completely overlapping chromosome clusters is poor. This type of method determines the intersection and concave point of the overlapping part of the chromosome by calculating the curvature and then performs segmentation. Therefore, the misjudgment and omission of the effective intersection point will seriously affect the performance of the segmentation.

Recently, more researches have constructed deep learning methods to accomplish medical image processing tasks, which can effectively avoid the occurrence of the aforementioned issues. Similar to natural image segmentation, chromosome segmentation methods based on deep learning are mainly divided into semantic segmentation (Shelhamer, et al., 2017) and instance segmentation (Fathi et al., 2017). As for chromosome semantic segmentation tasks, Hu et al. constructed the U-Net with two-layer pooling to segment overlapping chromosomes with less computation and storage costs (Hu, et al., 2017). The segmentation accuracy and Intersection over Union (IoU) score (McGuinness and O’Connor, 2010) for overlapping regions are 99.22 and 94.70, respectively, where the segmentation accuracy is high, but the IoU score still needs to be improved. Saleh et al. believed that the increase of pooling and convolution operation in the network was conducive to the extraction of more input feature information (Saleh, et al., 2019). Thus, they built three-layer pooling in U-Net (Ronneberger et al., 2015) to segment overlapping chromosomes, and the segmentation accuracy and IoU were slightly improved. However, the aforementioned two methods are only applicable to scenarios where chromosomes overlap in pairs. However, real chromosome overlapping is much more complicate than that. Thus, it is not that sufficient to apply the aforementioned two methods to real chromosome data sets. As for the chromosome instance segmentation tasks, Bai et al. first used U-Net to segment the foreground in the chromosome image, and then YOLO v3 (Joseph Redmon, 2018) was constructed to obtain the target detection box of each chromosome, which is followed by U-Net to segment single chromosomes from the detection boxes in the final (Bai, et al., 2020). The YOLO v3 backbone network used in this method is weak in detecting small targets and overlapping targets, so it does not work well in the scenarios that chromosomes overlap with each other severely. In addition, it disassembles the instance segmentation task into three networks, which makes the procedure cumbersome and inefficient.

It can be seen that the accuracy of the target detection box is extremely important in the chromosome instance segmentation tasks. Generally, when detecting clustered targets, the classification confidence of the target box is often high, but the actual detection result is poor, which leads to a decrease in the AP score with high IoU threshold. To address this issue, Jiang et al. constructed IoU Net, which predicts the IoU of the regression box and the ground truth box to replace original classification confidence, which eliminated the screening error caused by the misleading classification confidence, thus improved the target detection performance (Jiang et al., 2018). Wu et al. constructed the IoU-aware single-stage object detector. It also predicts the IoU of the regression box and the ground truth box and then uses it as a multiplicative operator to correct the classification confidence (Wu et al., 2020). The corrected confidence is better correlated with the positioning accuracy, which effectively improves the positioning accuracy. Chen et al. constructed the supervised edge attention network (SEA Net) (Chen et al., 2020). The IoU of the regression box and the ground truth box are achieved and multiplied with the classification confidence to improve the detection accuracy of the clustered target. Moreover, they designed an extra head branch to help predict the edge of mask to improve the segmentation effect when the IoU threshold is high. For instance, segmentation tasks where the classification confidence is high while the actual segmentation result is not that satisfactory, Huang et al. multiplies the IoU of the predicted mask and the ground truth mask with the classification confidence to construct the Mask Scoring RCNN (MS RCNN) (Huang, et al., 2019). It considers the classification score and the quality score of the predicted mask, and the segmentation result is further improved compared with Mask RCNN. The methods mentioned before adopted either the IoU of the predicted box or the IoU of the predicted mask, and the ground truth box to modify the classification confidence. However, it does not consider whether the prediction process is interpretable. If an interpretable method is adopted, the performance will be better.

### Contribution

This study proposes a chromosome instance segmentation network based on regression correction to achieve precise segmentation in the Giemsa-banding chromosome images. The main contributions of this study are summarized as follows.

1) Considering high classification confidence but poor detection and segmentation performance in reality, more relevant confidence of *P*_*Box*_ and *IoU*_*Mask*_ with positioning accuracy and segmentation accuracy are achieved without extra head branches to achieve better correction of the classification confidence. *P*_*Box*_ is the predicted probability based on the regression box, and *IoU*_*Mask*_ is the predicted IoU based on the mask.
2) Considering that the traditional non-maximum suppression algorithms based on the overlap screening of prediction boxes, which may result in missing or wrong target boxes, a non-maximum suppression algorithm based on instance mask screening is proposed to improve the segmentation of instances.
3) Since the traditional IoU loss function is not sensitive to the wrong segmentation area, *K*-IoU loss function is designed. It divides the area to be segmented into *K* parts and calculate the weight of each part to the overall segmentation loss according to the proportion of the area to be segment in each part to the total area, which improves the sensitivity of the network to error segmentation and makes the penalty reasonable.

## Methods

### Instance Segmentation Model Based on Regression Correction

**FIGURE 1:**
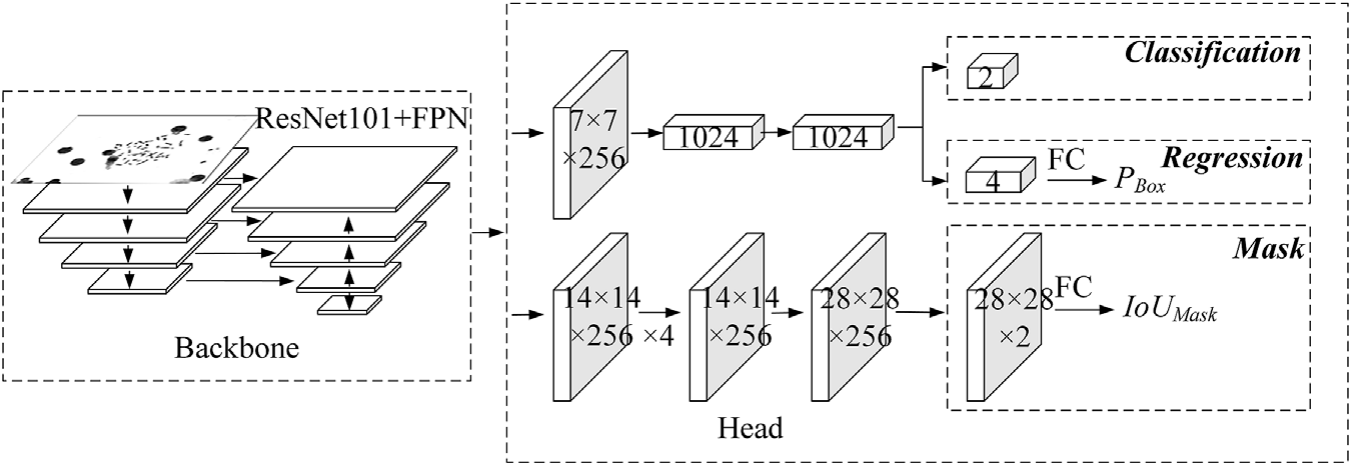
Structure of the regression correction network.

The multitask supervised learning method is known to make good use of valuable information to obtain more accurate results for each task. Its effectiveness lies in the correlation between all tasks. However, the predicted result of the regression branch is the offset of the regression box rather than the actual coordinates. There is no direct correlation between the offset and the IoU score, which makes it not reasonable enough. In addition, the use of IoU score to modify the classification confidence will cause the drop of classification confidence, thus worsen the subsequent non-maximum suppression operations. Therefore, Wu et al. and Chen et al. proposed regression branches to predict IoU scores under a multitask supervised learning framework, but the results showed low correlation with the real IoU scores. To address this issue, we propose here a regression correction-based instance segmentation network for chromosome segmentation, as shown in Figure 1.

First, a regression confidence *P*_*Box*_ is introduced, as shown in Eq. 1. Taking the prediction result of the regression branch as input, *P*_*Box*_ is predicted through a fully connected layer with 1,024 output nodes. It helps make the prediction process of *P*_*Box*_ more reasonable, which shows stronger correlation with positioning accuracy.

$$P_{B\text{ox}}=1-\left(T\left(L_{Reg}\right)\right)$$ (1)

where *T*(·) is tanh function, and *L*_*Reg*_ is the regression loss, which is calculated by the Smooth L1 loss function.

Due to the direct correlation between the output of the Mask branch and *IoU*_*Mask*_, the output of the mask branch acts as input, and the *IoU*_*Mask*_ is predicted by the fully connected layer with 1,024 output nodes, instead of multitask supervised learning, which helps make the prediction process more interpretable, as shown in Figure 1.

Finally, the regression confidence *P*_*Box*_, as well as *IoU*_*Mask*_, which is more relevant with the segmentation accuracy, are used to correct the classification confidence. Thus, both the detection score and the segmentation score are considered simultaneously to achieve better instance segmentation performance.

### Mask-Based Non-Maximum Suppression Algorithm

For overlapping target detection, the non-maximum suppression algorithm should be further improved due to its poor effect on severe overlapping (Neubeck and Van Gool, 2006). Therefore, Bodla et al. proposed a Soft-NMS algorithm, which weakens the lower confidence of the overlapping detection box by multiplying it by a weight, instead of directly discarding it (Bodla, et al., 2017). The detection performance of overlapping targets is slightly improved, while the time complexity significantly increased. A Box-based non-maximum suppression algorithm is beneficial to target detection tasks. However, the effect is general in the instance segmentation task. As shown in Supplementary Figure S2, both boxes are the prediction boxes of the two chromosomes, respectively, and the IoU of the two boxes is 0.8. Thus, the overlapping is severe. Following conventional processing, boxes with higher classification confidence will be remained, while boxes with lower classification confidence will be discarded, resulting in missing detection of target boxes in this case. However, the analysis found that the IoU of the mask was only 0.2 at this time, which was much lower than the IoU of the detection boxes.

Therefore, a mask-based non-maximum suppression algorithm is proposed here for overlapping chromosome segmentation tasks. The algorithm aims to remain as many prediction boxes as possible before the prediction box fed into the mask branch and then calculates the IoU of each prediction mask and other prediction masks. Finally, traverse the classification confidence from high to low and remove prediction masks that have an IoU score greater than that of the threshold with the current prediction mask. It makes use of the IoU of the mask as a threshold to help select overlapping targets, which can effectively prevent missing and misjudged overlapping targets, thus improve segmentation performance.

### *K*-IoU Loss Function

There are multiple metrics for segmentation performance evaluation. Among them, IoU is the most widely used one, and better segmentation performance expects higher IoU score. Thus, the IoU loss function (Yu, et al., 2016) is often used for model parameter optimization, as shown in Eq. 2.

$$L_{IoU}=-\ln IoU_{Mask},$$ (2)

where *IoU*_*Mask*_ represents the IoU score between the predicted mask and its ground truth.

**FIGURE 2:**
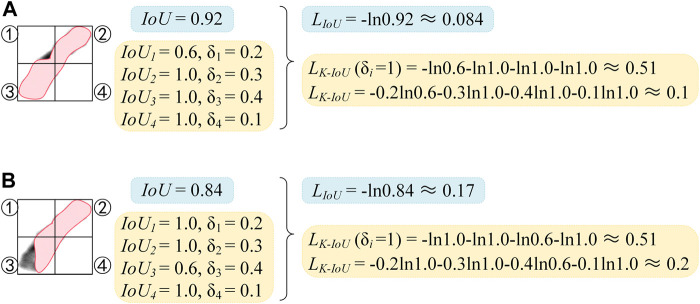
Comparison diagram of *L*_*IoU*_ and *L*_*K-IoU*_. **(A)** Calculation of LIoU. **(B)** Calculation of L_K-IoU_.

However, IoU can only represent the overall segmentation quality of the prediction results. It cannot adequately represent the segmentation quality of some key regions. Under chromosome segmentation scenarios that chromosomes exhibit variable shapes, fuzzy edges, and severe overlaps, the difficult-to-segment regions are the key regions that call for more attention. The segmentation quality of key regions may better help karyotypists to diagnose, thus provides more reliable information for physicians’ choice of medical regime. Thus, a more effective and reasonable loss function, *L*_*K-IoU*_, is proposed for the incorrectly segmented region, as shown in Eq. 3. By minimizing the *K*-IoU loss function, the network has better segmentation performance for difficult-to-segment regions.

$$L_{K-IoU}\;\;=\;\;-\sum_{i=1}^{K}\delta_{i}\ln IoU_{i},$$ (3)

$$\delta_{i}=\frac{Mask_{i}}{Mask},$$ (4)

where *K* indicates the number of different parts that the ground truth mask is divided into. As shown in Figure 2, *K* is 4 and the shape is 2 × 2, the ground truth mask is equally divided by two vertical center lines to obtain four parts. As shown in Eq. 4, *δ*_*i*_ indicates the proportion of the ground truth in the *i*-part over the entire ground truth, and *IoU*_*i*_ indicates the IoU of the predicted mask and the ground truth in the *i*-part.

As shown in Figure 2, the chromosome is divided into four parts, which are indicated as ①, ②, ③, and ④. The IoU scores and *δ*_*i*_ scores of the four parts are demonstrated, with the striped area being the predicted mask. In Figure 2A, except for the lower IoU score in part ①, the IoU scores of all the other parts are 1. Suppose the conventional IoU loss function is used, the high IoU scores of the other three parts will weaken the negative impact caused by the incorrect segmentation in the first part and reduce the sensitivity of the network to the incorrect segmentation. Finally, the loss of 0.084 can be achieved. In contrast, a loss of 0.51 can be obtained if the *L*_*K-IoU*_ (*δ*_*i*_ = 1) is used. Compared with the IoU loss function, better segmentation performance can be obtained when the loss converges to the same value, and the sensitivity of the network to incorrect segmentation is dramatically improved.

However, it is not necessary to blindly increase the sensitivity of the network to incorrect segmentation. When the proportion of the ground truth mask in a certain part to the entire ground truth mask becomes lower, the influence of this part on the whole is smaller. Comparing Figure 2A with Figure 2B, the segmentation result in (a) is significantly better than that in (b), but their *L*_*K-IoU*_ (*δ*_*i*_ = 1) are the same. It may thank *L*_*K-IoU*_ that corrects the loss of each part through the weight *δ*_*i*_, as shown in Figure 2B. It is more sensitive to incorrect segmentation and can better highlight the contribution of critical areas to loss.

Then, we define the multitask loss on each proposal as the sum of the losses from Box head and Seg head, as shown in Eq. 5.

$$L=L_{Box}+L_{Seg},$$ (5)

where *L*_*Box*_ is composed of three parts, which are defined in Eq. 6.

$$L_{Box}=L_{Cls}+L_{Reg}+L_{P_{Box}},$$ (6)

where *L*_*Cls*_ is calculated by the cross-entropy loss function, and $L_{P_{Box}}$ is calculated by the cross-entropy loss function based on *P*_*Box*_ obtained by Eq. 1.

*L*_*Seg*_ is also composed of three parts:

$$L_{Seg}=L_{Mask}+L_{K-IoU}+L_{IoU_{Mask}},$$ (7)

where *L*_*Mask*_ is the binarized cross-entropy loss function, and *L*_*K-IoU*_, calculated by Eq. 3, is also the binarized cross-entropy loss.

## Experimental Results

Wein the next conducted five-fold cross-validation experiments on 985 real chromosome Giemsa-banding chromosome images of 1,600 × 1,200 pixels. A total of 60% of the data was allocated for training, while the remaining 40% images were equally partitioned and referred to as validation and test sets. These images were first scaled and padded to 512 × 512 and data augmentation was also involved to better train the models.

Mask RCNN (He, et al., 2017), PANet (Liu, et al., 2018), IoU Net, and MS RCNN with different backbone network were compared on the same dataset. The hyperparameters of the model proposed in this study follow Mask RCNN. The initial learning rate is 1e-5, the learning momentum is 0.9, and the weight decay is 0.0001. Due to the hardware limitations and image size, the batch size is set to 1, and stochastic gradient descent (SGD) is used for training for 100 epochs.

### Evaluation Metrics

For the evaluation of target detection, AP^M^ (Lin et al., 2014) is adopted in this study. AP^M^ represents the average accuracy value of mask’s IoU threshold from 0.5 to 0.95 with an interval of 0.05. AP^M^_50_ refers to the AP^M^ score with mask IoU threshold being 0.5, while AP^M^_75_ refers to the score with mask IoU threshold being 0.75.

### Main Result

As shown in Table 1, our proposed method achieves stable improvements on different models and backbone networks. With ResNet 101 + FPN, the AP^M^ of Mask RCNN+RC reaches 83.35%, with an increase of 3.76%. Since PANet follows the hyperparameters of Mask RCNN, the segmentation results of PANet are not as good as Mask RCNN, but when the backbone network is ResNet101 + FPN, the AP^M^ is still significantly improved with an increase of 2.64%.

**TABLE 1:** Performance comparison of different network models.

Baseline	Backbone	AP^M^		${\text{AP}}_{50}^{\text{M}}$		${\text{AP}}_{75}^{\text{M}}$	
		—	+RC^a^	—	+RC	—	+RC
Mask RCNN	ResNet 101 + FPN	**79.59**	**83.35**	**97.94**	**99.13**	**96.75**	**98.06**
	ResNet 50 + FPN	72.22	74.72	96.87	97.61	90.86	93.38
PANet	ResNet 101 + FPN	76.56	79.2	98.47	98.57	95.34	96.09
	ResNet 50 + FPN	70.82	73.2	96.17	97.43	90.17	91.55

a +RC represents adding the methods proposed in this article on the basis of the baseline. Best results are indicated in Bold.

## Discussion

Performance of regression correction network: compared with the baseline Mask RCNN, the chromosome instance segmentation network based on regression correction in this study can significantly improve the accuracy of instance segmentation and enhance the AP^M^ score by 3.76%, as shown in Table 2. Experimental results show that introducing a mask-based non-maximum suppression algorithm is effective for improving the performance of instance segmentation. As shown in Supplementary Figure S3, the left image presents the segmentation result of the baseline model Mask RCNN, the right one displays the segmentation result of the mask-based non-maximum suppression algorithm assembled on the baseline model, and the weights of the two models are the same. It can be seen that the mask-based non-maximum suppression algorithm effectively prevents the omission of segmentation masks without training.

**TABLE 2:** Ablation experiment results.

Baseline	M-NMS	K-IoU	*IoU*_*Mask*_	*P*_*Box*_	*IoU*_*Box*_	AP^M^	${\text{AP}}_{50}^{\text{M}}$	${\text{AP}}_{75}^{\text{M}}$
Mask RCNN^a^						79.59	97.94	96.75
MS RCNN	√		√			80.58	99.07	97.66
IoU Net	√				√	80.31	99.16	97.57
RC-Net (ResNet101 + FPN)	√					80.38	98.16	97.64
	√	√				80.85	99.08	97.87
	√		√			81.56	99.27	97.94
	√			√		81.97	99.09	97.90
	√	√	√	√		**83.11**	**99.09**	**98.05**

a The second row is the baseline Mask RCNN framework. The component with √ is added to the baseline. Best results are indicated in Bold.

In the meanwhile, the introduction of the *K*-IoU loss function helps improve the sensitivity to incorrect segmentation. It not only strengthens the penalty for incorrect segmentation but also considers the proportion of segmentation errors, on the whole, making the penalty more reasonable. Therefore, AP^M^ is further improved. In this study, the grid search method is used to determine the value of *K*. As shown in Supplementary Table S1, when *K* is 4, the AP^M^ score is the highest, and when the *K* is further increased, the AP^M^ score decreases. Therefore, this study sets the value of *K* to 4. Analyzing the reason, when the shape is refined, the IoU of the prediction mask and the ground truth mask in some grids will be 0, resulting in the back-propagation gradient being 0, and optimization training cannot be performed.

By comparing the method of directly using the output of the Mask branch to predict *IoU*_*Mask*_ (the seventh row) and the method of MS RCNN, both use the predicted *IoU*_*Mask*_ to correct the classification confidence. The segmentation performance of the former is better than that of MS RCNN. This verifies from the side that the method in this study makes the predicted correlation between *IoU*_*Mask*_ and Mask stronger and is more helpful to correct classification confidence.

The IoU Net–based method, which uses *IoU*_*Box*_ instead of classification confidence, is ineffective and even leads to a decrease in AP^M^. It is due to the fact that the correlation between the output of the regression branch and *IoU*_*Box*_ is not strong enough. Therefore, this article uses a more relevant head branch to predict the regression confidence *P*_*Box*_ to correct the classification confidence (the eighth row). Compared with IoU Net, AP^M^ has a more significant improvement, which means that the regression confidence *P*_*Box*_ can modify the positioning accuracy of the prediction box more than *IoU*_*Box*_. Finally, this study considers both the positioning accuracy of the prediction box and the segmentation accuracy of the instance (the ninth row). The AP^M^ has been further improved to 83.11 with an increase of 2.73%.

The design of confidence weight: This study considers the positioning accuracy *P*_*Box*_ of the prediction box and segmentation accuracy *IoU*_*Mask*_ of the instance at the same time to improve the segmentation performance of the network. However, multiplying the two directly with the classification confidence may not be the best choice. Therefore, *P*_*Box*_ and *IoU*_*Mask*_ are exponentiated, and the AP^M^ scores obtained are shown in Supplementary Table S2. When *IoU*_*Mask*_ is “√2” and *P*_*Box*_ is “√,” the specific calculation method is shown in Eq. 8.

$$P_{Cls}=P_{Cls}\cdot IoU_{Mask}^{2}\cdot P_{Box},$$ (8)

We can see that when *P*_*Box*_ is calculated to the sixth power, AP^M^ reaches the highest score of 83.35%. Moreover, the improvement is more significant than the effect brought by the exponentiation of *IoU*_*Mask*_. It can be seen that AP^M^ is more sensitive to *P*_*Box*_, further verifying the effectiveness of *P*_*Box*_.

## Conclusion

This article focuses on improving the segmentation accuracy of chromosome instances in real chromosome datasets, significantly overlapping chromosomes. We respectively use the output of the regression branch and the mask branch to predict two confidences, *P*_*Box*_ and *IoU*_*Mask*_, which are more relevant to the positioning accuracy and segmentation accuracy and achieve a better correction of the classification confidence. A non-maximum suppression algorithm based on mask is proposed, which uses the overlap of the instance as the basis for judgment, which effectively prevents the missing and incorrect segmentation of the chromosomes. Moreover, a *K*-IoU loss function is proposed, which improves the network’s sensitivity to incorrect segmentation while fully considering the impact of the incorrect segmentation on the whole so that the penalty is reasonable. The experimental results show that the method in this study greatly improves the accuracy of instance segmentation on the baseline Mask RCNN, and it also has a good effect on PANet. Since the implementation of *P*_*Box*_ and *IoU*_*Mask*_ does not require additional head branches and the structure is relatively simple, it is expected to be extended to other models which aim at instance segmentation.

## Data Availability

The raw data supporting the conclusions of this article will be made available by the authors, without undue reservation.
