# Supplementary material for: RC-Net: Regression Correction for End-To-End Chromosome Instance Segmentation
Source: Front Genet. 2022 May 18;13:895099. doi: 10.3389/fgene.2022.895099 (PMC9158129; doi:10.3389/fgene.2022.895099)
Supplement: Supplementary file 1 [file Table1.docx]

**Table S1 Performance comparison of different *K* values.**

| K | Shape | AP^M^ | $\mathrm{AP}_{50}^{\mathrm{M}}$ | $\mathrm{AP}_{75}^{\mathrm{M}}$ |
| --- | --- | --- | --- | --- |
| 1 | 1×1 | 80.38 | 98.16 | 97.64 |
| 2 | 1×2 | 80.53 | 98.76 | 97.77 |
| 2 | 2×1 | 80.49 | 98.72 | 97.71 |
| 4 | 2×2 | **80.85** | **99.08** | **97.87** |
| 9 | 3×3 | 80.79 | 98.88 | 97.81 |

Best results are indicated in Bold.
