# Supplementary figures and images for: RC-Net: Regression Correction for End-To-End Chromosome Instance Segmentation
Source: Front Genet. 2022 May 18;13:895099. doi: 10.3389/fgene.2022.895099 (PMC9158129; doi:10.3389/fgene.2022.895099)

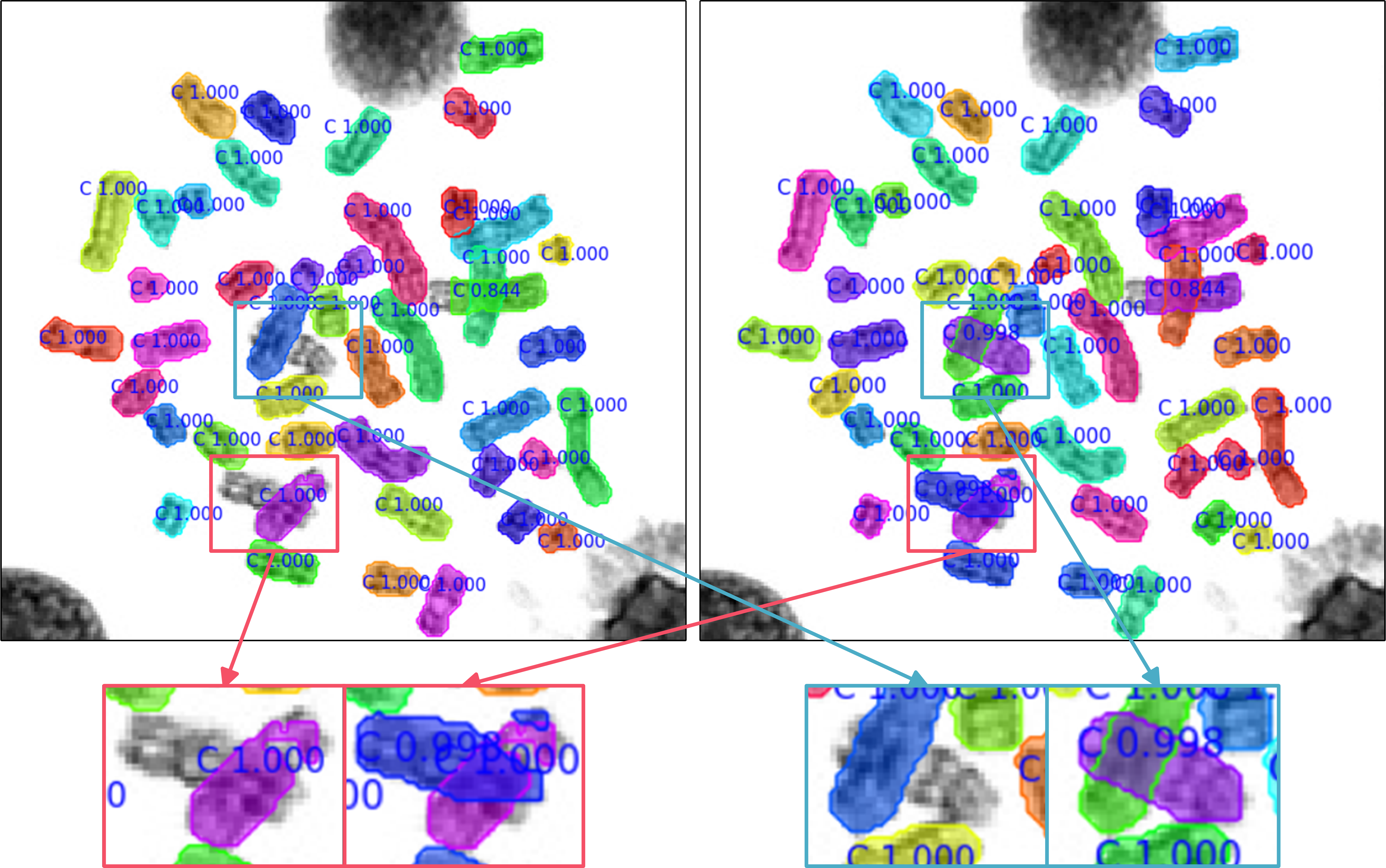

Supplement: Supplementary file 2 [file Image3.TIF]

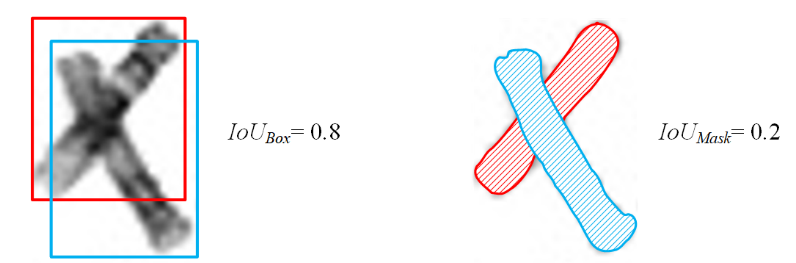

Supplement: Supplementary file 3 [file Image2.TIF]

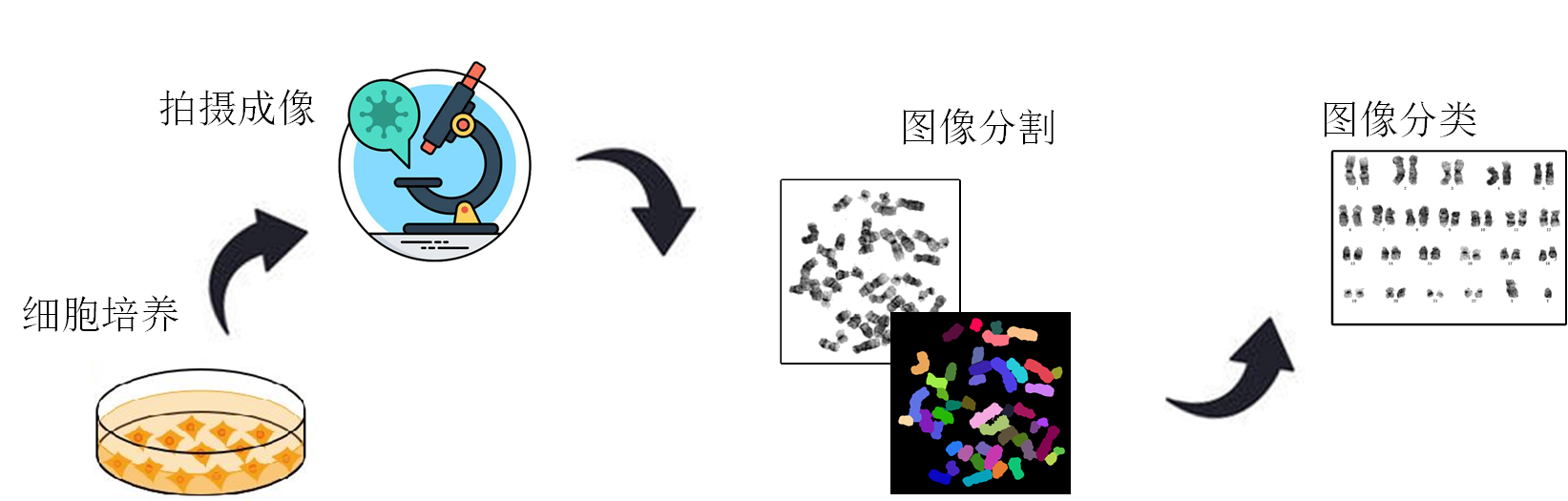

Supplement: Supplementary file 4 [file Image1.TIF]
