# Supplementary material for: RC-Net: Regression Correction for End-To-End Chromosome Instance Segmentation
Source: Front Genet. 2022 May 18;13:895099. doi: 10.3389/fgene.2022.895099 (PMC9158129; doi:10.3389/fgene.2022.895099)
Supplement: Supplementary file 5 [file Table2.docx]

**Table S2 Target detection results with different confidence weight.**

| Method | *IoU_Mask_* | *P_Box_* | AP^M^ | $\mathrm{AP}_{50}^{\mathrm{M}}$ | $\mathrm{AP}_{75}^{\mathrm{M}}$ |
| --- | --- | --- | --- | --- | --- |
| RC Net  (ResNet101+FPN) | √ | √ | 83.11 | 99.09 | 98.05 |
|  | √2^*^ | √ | 83.15 | 99.07 | 98.03 |
|  | √3 | √ | 83.09 | 99.03 | 97.99 |
|  | √4 | √ | 82.99 | 98.99 | 97.94 |
|  | √5 | √ | 82.93 | 98.97 | 97.91 |
|  | √ | √2 | 83.24 | 99.11 | 98.07 |
|  | √ | √3 | 83.27 | 99.13 | 98.07 |
|  | √ | √4 | 83.30 | 99.13 | 98.09 |
|  | √ | √5 | 83.32 | 99.13 | 98.08 |
|  | √ | √6 | **83.35** | **99.13** | **98.09** |
|  | √ | √7 | 83.35 | 99.13 | 98.06 |
|  | √ | √8 | 83.35 | 99.13 | 98.06 |
|  | √ | √9 | 83.32 | 99.12 | 98.04 |

^*^√2 represents taking the second power of *IoU_Mask_* or *P_Box_*, √3 represents taking the third power of *IoU_Mask_* and *P_Box_*, and so on. Best results are indicated in Bold.
